# Guillain‐Barré syndrome: a comprehensive review

**DOI:** 10.1111/ene.16365

**Published:** 2024-05-30

**Authors:** Roberto Bellanti, Simon Rinaldi

**Affiliations:** ^1^ Nuffield Department of Clinical Neurosciences University of Oxford Oxford UK

**Keywords:** diagnosis, Guillain‐Barré syndrome, immunopathology, prognosis, treatment

## Abstract

Guillain‐Barré syndrome (GBS) is a potentially devastating yet treatable disorder. A classically postinfectious, immune‐mediated, monophasic polyradiculoneuropathy, it is the leading global cause of acquired neuromuscular paralysis. In most cases, the immunopathological process driving nerve injury is ill‐defined. Diagnosis of GBS relies on clinical features, supported by laboratory findings and electrophysiology. Although previously divided into primary demyelinating or axonal variants, this dichotomy is increasingly challenged, and is not endorsed by the recent European Academy of Neurology (EAN)/Peripheral Nerve Society (PNS) guidelines. Intravenous immunoglobulin and plasma exchange remain the primary modalities of treatment, regardless of the electrophysiological subtype. Most patients recover, but approximately one‐third require mechanical ventilation, and 5% die. Disease activity and treatment response are currently monitored through interval neurological examination and outcome measures, and the potential role of fluid biomarkers is under ongoing scrutiny. Novel potential therapies for GBS are being explored but none have yet modified clinical practice. This review provides a comprehensive update on the pathological and clinical aspects of GBS for clinicians and scientists.

## INTRODUCTION

Guillain‐Barré syndrome (GBS) is the most common cause of acute neuromuscular paralysis worldwide. It can be a severe and life‐threatening condition, and early treatment is essential for a better prognosis. Over 100 years after it was first described [1], GBS is now one of the best understood neuroinflammatory diseases, providing valuable insights into the mechanisms of peripheral nerve inflammation. However, many aspects of its nature continue to elude our comprehension, and the full scope of GBS remains a puzzle yet to be fully deciphered. We know that GBS is a postinfectious, monophasic, immune‐mediated polyradiculoneuropathy, and its diagnosis is largely based on clinical patterns with or without the support of laboratory findings and electrophysiology. About a third of patients with GBS develop a severe, generalised neuropathy, and ultimately require mechanical ventilation due to respiratory failure. One in 20 patients die. Increased understanding of GBS has not been paralleled by progress in therapeutics, and the mainstay of treatment remains intravenous immunoglobulin (IVIg) or plasma exchange (PLEX). Outcome measures and interval neurological examination are used to monitor disease activity and response to treatment, whilst the potential role of fluid biomarkers of neuropathy remains under evaluation. Novel therapies are also being explored and may soon contribute to clinical management. In light of the new European Academy of Neurology (EAN) and Peripheral Nerve Society (PNS) guidelines on diagnosis and treatment of GBS [2], this review summarises current knowledge and best available evidence, to inform clinicians and scientists as they navigate the journey through Guillain‐Barré syndrome.

## EPIDEMIOLOGY

There are 100,000 new cases of GBS every year [3]. Estimates of yearly incidence (per 100,000 people) are lowest in Japan (0.44) [4], China (0.67) [5], Tanzania (0.83) [6], and Finland (0.84) [7], and highest in Chile (2.12) [8] and Bangladesh (3.25) [9], likely due to differences in exposure to infectious organisms. Seasonal variations are described [10], and spikes of GBS have been reported following infectious outbreaks, most notably in relation to *Campylobacter jejuni* [11] and Zika virus (ZIKV) [12, 13]. Older people are more commonly affected (peak incidence of GBS is between 50 and 70 years of age) and the male:female ratio is 1.5:1 [3, 14, 15].

## PATHOPHYSIOLOGY OF GBS

The pathophysiology of GBS can be delineated into two pivotal stages: initiation by an immunological trigger and immune‐mediated disruption of axons and/or myelin. Based on electrophysiology, GBS has been traditionally divided into two forms: acute inflammatory demyelinating polyradiculoneuropathy (AIDP) and acute motor axonal neuropathy (AMAN). The notion that this neurophysiological dichotomy reflects a true underlying pathological difference between primarily demyelinating versus axonal GBS is currently being challenged, and the new EAN/PNS guidelines no longer support the distinction between AIDP and AMAN.

### Disease initiation

#### Prodromal infections

**FIGURE 1:**
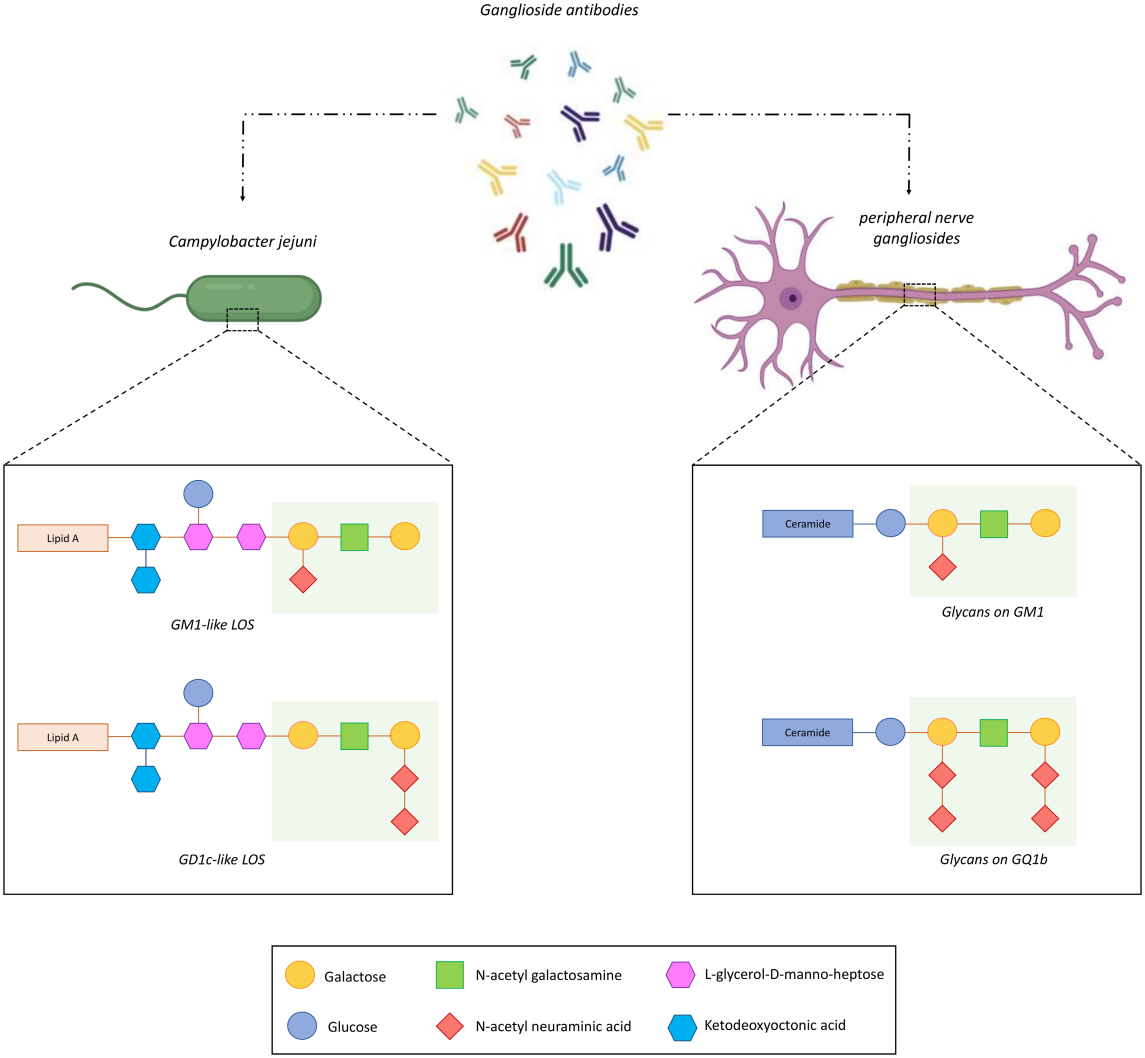
Lipo‐oligosaccharide (LOS) on the outer membrane of *Campylobacter jejuni* induces cross‐reactive antibodies which, through molecular mimicry, bind to the structurally identical glycans (areas in green) present on peripheral nerve gangliosides (GM1 and GQ1b in the example above), resulting in damage to axons and Schwann cells. [Correction added on 03 June 2024, after first online publication: Figure 1 has been corrected.]

In most cases, GBS is a postinfectious disease, and two‐thirds of patients report prodromal gastrointestinal or respiratory symptoms. *Campylobacter jejuni*, the most commonly identified pathogenic trigger, leads to GBS in approximately 1 out of every 1000 cases, through molecular mimicry between its surface lipo‐oligosaccharide (LOS) and host peripheral nerve gangliosides (Figure 1). This triggers the production of cross‐reactive antibodies targeting gangliosides like GM1, GD1a, and GQ1b, resulting in axoglial damage. Other pathogens associated with GBS include Epstein–Barr virus (EBV), cytomegalovirus (CMV), hepatitis E virus (HEV), *Mycoplasma pneumoniae*, *Haemophilus influenzae*, influenza A virus, and Zika virus. *Mycoplasma pneumoniae* is associated with anti‐galactocerebroside antibodies of the IgG isotype, more frequently in children [16, 17]. The mechanistic association between most of these pathogens and axoglial damage remains unclear, and molecular mimicry between *C. jejuni* and peripheral nerve glycans is still the best explained postinfectious link in GBS, although how patients break tolerance to self‐glycans following *C. jejuni* infection has not been clarified.

#### Vaccines associated with GBS

Epidemiological evidence links some vaccines with a subsequent diagnosis of GBS. These include the vaccine for the ‘swine flu’ (A/New Jersey/76 influenza), the recombinant zoster vaccine (RZV), and the adenovirus‐vector SARS‐CoV‐2 vaccines. The ‘swine flu’ vaccine campaign in the USA was associated with 4.9–5.9 GBS cases per million vaccinations [18]. Though initial concerns were raised about the H1N1 influenza A vaccination in 2009, subsequent surveillance reported only 1–1.6 cases per million doses [19, 20, 21]. Similarly, herpes zoster vaccination with RZV showed a marginally increased risk of GBS, with approximately 3 excess cases per million vaccinations [22]. Adenovirus‐vectored SARS‐CoV‐2 vaccines, such as ChAdOx1 and Janssen COVID‐19 vaccines, have been linked to around 5.7 excess GBS cases per million first doses [23, 24, 25]. The 2023 EAN/PNS GBS guidelines conclude that the advantages of vaccination (reduction in morbidity and mortality related to infection and infection‐associated GBS) significantly outweigh any marginal elevation in the risk of post‐vaccine GBS [2]. It remains uncertain whether repeat or future vaccination is safe following presumed vaccine‐associated GBS, and certain individuals may be more susceptible to autoinflammation due to genetic predisposition. The risk of post‐vaccine GBS should be acknowledged and balanced against the benefits of vaccination overall.

#### Other proposed or established causal associations

The risk of developing GBS may be higher during the first 6 weeks after surgery [26], most significantly orthopaedic or gastrointestinal [27], and particularly in the context of active malignancy [28]. Cases of GBS in stem cell transplant patients immunosuppressed with tacrolimus for graft versus host disease (GVHD) prophylaxis have been reported [29], as well as in patients treated with immune checkpoint inhibitors [30]; however, the actual risk has not been ascertained in controlled studies.

### Immune‐mediated axoglial disruption

The inflammatory nature of GBS was first acknowledged in the late 1940s, when prominent lymphocyte infiltration was found in post‐mortem nerve biopsies [31]. It is now clear that both innate and cellular immune mechanisms are implicated in GBS, and T cells, B cells, NK cells, dendritic cells, and macrophages are all likely to contribute to axonal damage and demyelination.

**FIGURE 2:**
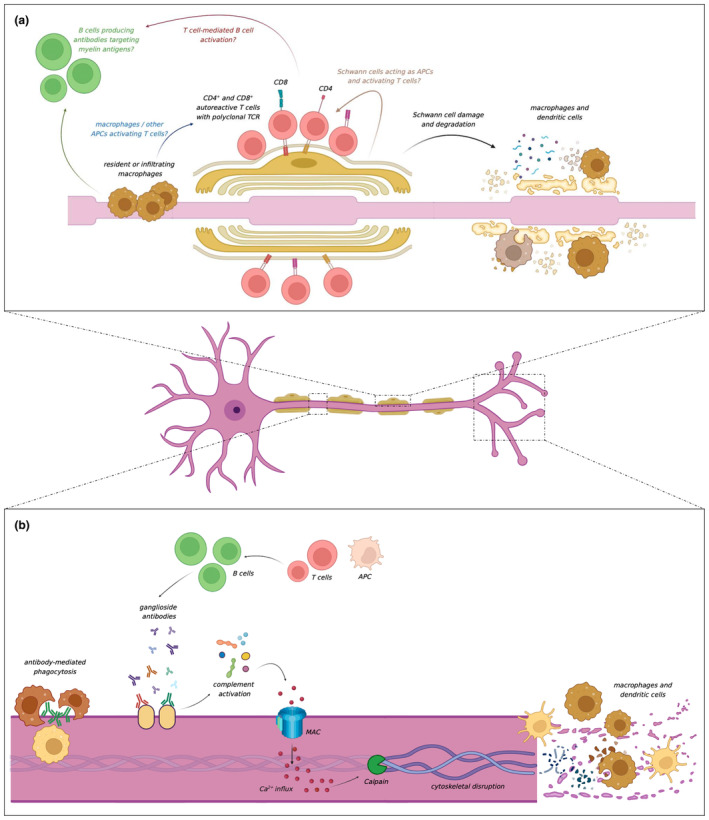
(a) T cells reactive to peripheral nerve myelin antigens. A humoral mechanism underlying primary glial damage has long been hypothesised, but anti‐myelin antibodies have yet to be identified. It remains unclear whether T cells are activated by macrophages (or other antigen‐presenting cells [APCs]), or by the Schwann cells themselves. (b) Ganglioside antibody binding leads to complement activation, membrane attack complex (MAC) formation, calcium influx into the axon, calpain‐mediated cytoskeletal disruption, and ultimately damage to the node of Ranvier, the paranode, and the motor nerve terminals. Dendritic cells, macrophages, and other phagocytes are also typically found within the nerve in patients with Guillain‐Barré syndrome. [Correction added on 03 June 2024, after first online publication: Figure 2 has been corrected.]

A recent study investigating autoreactive T cell immunity in patients with GBS [32] found that patients with AIDP had CD4+ and CD8+ T cells in their blood, cerebrospinal fluid (CSF) and nerve tissue reactive to myelin protein 0 (P0), myelin protein 2 (P2), or peripheral myelin protein 22 (PMP22) (Figure 2a). Autoreactive memory CD4+ T cells showed a proinflammatory cytotoxic T_H_1‐like phenotype and expressed genes with a known autoimmunity association. A similar T cell autoreactivity was not found in patients with AMAN, suggesting that patients with demyelinating and axonal GBS may actually have distinct immunopathological mechanisms. Whether antibody‐mediated primary Schwann cell damage occurs, and the potential contribution of T cells to B cell activation, remains to be definitively established.

Animal models [33, 34, 35, 36, 37, 38] using active immunisation or passive transfer have shown that axoglial damage can be mediated by ganglioside antibodies. In these models, autoantibodies target axonal or glial antigens and cause damage through complement activation and formation of the membrane attack complex (MAC) or induce non‐complement‐mediated effects on myelin and axonal regeneration. Complement‐dependent membrane disruption leads to aberrant influx of calcium into the axon, calpain activation, and ultimately damage to the node of Ranvier, the paranode, and the motor nerve terminals (Figure 2b) [35, 36, 39, 40]. Ganglioside antibody binding also leads to displacement of voltage‐gated sodium channels, cytoskeletal anchoring proteins, and cell adhesion molecules within the nodal and paranodal regions [38]. In transgenic mice where GM1 is only expressed by Schwann cells and not axons, GM1 antibodies cause primary glial injury. However, following Schwann cell/myelin dehiscence, axonal nanoruptures are formed, ultimately resulting in a convergence of the pathological process with influx of calcium, calpain activation within the axon, and cleavage of cytoskeletal and transmembrane proteins [41]. Such findings suggest that regardless of whether the initial injury is axonal or glial, there is a common final pathway which leads to axonal disruption and functional failure.

Ultimately, nerve conduction failure and the related spectrum of neurological symptoms seen in patients with GBS are due to damage to neurons (node, paranode, juxtaparanode, terminal axons) or glia (myelin, Schwann cells) or both. These alterations then lead to reversible conduction failure with the potential for rapid recovery, demyelination with slower recovery, or axonal degeneration with protracted and more often incomplete recovery, or a combination of all three within the same nerve [38].

At least 40% of GBS patients do not have identifiable serum or CSF autoantibodies. This may be because the full range of relevant antigenic targets have yet to be found. Glycolipid domains can associate to form complex hetero‐dimeric clusters, and around half of GBS patients have antibodies targeting these complexes but not their individual components in isolation [42, 43]. Furthermore, the pathogenic potential of certain antibodies is determined by local interaction of glycolipids with neighbouring gangliosides, which can prevent binding of autoantibodies.

Genetic factors might also play a role in the immunobiology of GBS. A metanalysis found a moderate association between GBS and a tumour necrosis factor (TNF) gene polymorphism [44]. An association has also been found between GBS severity and polymorphism of the mannose‐binding lectin (MBL) gene [45], which contributes to the activation of the complement system and to the clearance of apoptotic cells by macrophages and dendritic cells. Genetic studies on large GBS cohorts have yet to be conducted, and genome‐wide association studies in large, deeply phenotyped cohorts are needed to determine whether and to what extent GBS is also genetically determined.

## CLINICAL FEATURES OF GBS AND DIAGNOSTIC CRITERIA

### Clinical features

**FIGURE 3:**
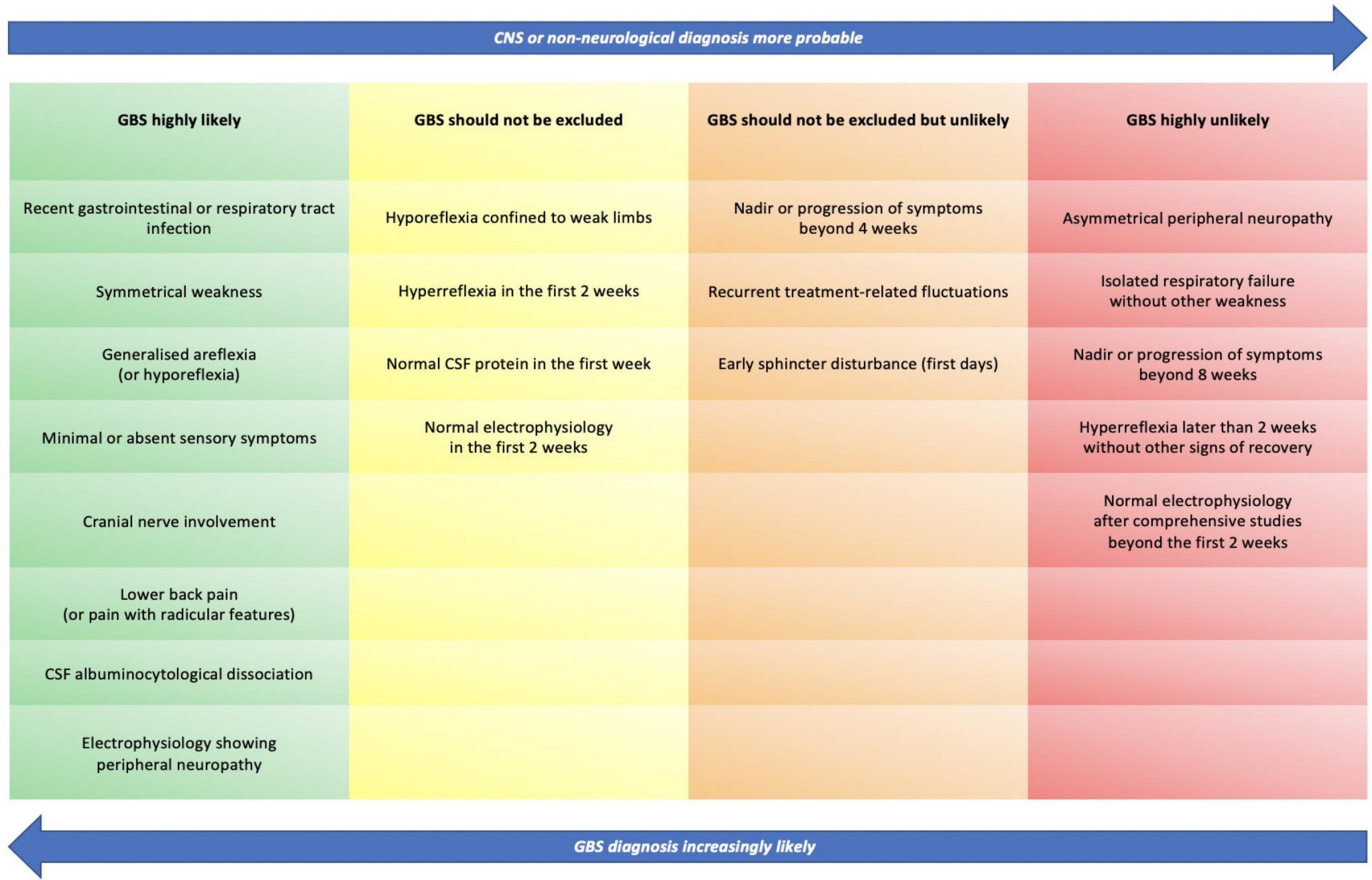
Clinical features that support or refute a diagnosis of Guillain‐Barré syndrome (GBS). CNS, central nervous system; CSF, cerebrospinal fluid.

GBS presents with acute, rapidly progressive flaccid paralysis of arms and legs, and absent or decreased deep tendon reflexes (Table 1). Sensory disturbance may or may not be present. Large studies have shown that, typically, patients reach nadir of disability within 10–14 days, and symptoms rarely progress beyond 4 weeks [15, 46, 47]. CSF albuminocytological dissociation, with high protein level and normal white cell count (WCC), is a hallmark of GBS and, together with raised CSF/serum albumin quotient (Qalb), indicates disruption of the blood–nerve barrier in the affected nerve roots, whereby proteins exude into the subarachnoid space through the leaky vessels. Normal CSF protein is common during the first week of the disease and does not exclude GBS, and may be normal or only mildly elevated in the second week after onset of symptoms. Higher WCC should raise suspicion for infections (HIV, Lyme), haematological malignancy, or granulomatous inflammatory disorders such as sarcoidosis. Crucially, CSF should be sampled prior to treatment with IVIg, which typically raises both CSF protein and WCC. Nerve conduction studies and electromyography may be normal during the first days, or show inexcitable nerves, indicating severe nerve damage, but are almost never normal weeks after disease onset. Similarly, initial hyperreflexia and sphincter disturbance (bladder and/or bowel dysfunction), though unusual and raising the suspicion of an alternative diagnosis, do not exclude GBS (Figure 3). Encephalopathy or altered level of consciousness may suggest Bickerstaff brainstem encephalitis (BBE), and a sensory level likely favours a myelopathy. However, involvement of the thoracic roots or intercostal nerves may occasionally produce a pseudo‐spinal level in GBS. In the latter case, length‐dependent involvement may produce an anterior “level” that is not reproduced posteriorly. Finally, recurrent clinical worsening after initial improvement or stabilisation, known as treatment‐related fluctuations (TRF), may occur in some patients with GBS if duration of the disease outlasts duration of action of therapy. However, three or more TRFs and/or fluctuations after 8 weeks suggest that the diagnosis is *not* GBS, and alternative inflammatory neuropathies such as chronic inflammatory demyelinating polyradiculoneuropathy (CIDP) or an autoimmune nodopathy (AIN) should be considered. The phenotypical spectrum of GBS is summarised in Table 2.

**TABLE 1:** Guillain‐Barré syndrome GBS diagnostic criteria (2023 European Academy of Neurology [EAN]/Peripheral Nerve Society [PNS] guidelines).

Essential criteria
Progressive weakness of upper and lower limbs
Absent or decreased deep tendon reflexes
Progression of clinical worsening not beyond 4 weeks
Clinical features supportive of GBS
Symmetrical neuropathy
Mild or absent sensory symptoms (compared to motor)
Cranial nerve involvement (especially bilateral facial palsy)
Respiratory failure
Autonomic dysfunction
Recent gastrointestinal or respiratory infection (<6 weeks)
Back pain (interscapular or radicular)
Other supportive findings
Cerebrospinal fluid albuminocytological dissociation
Electrophysiology confirming peripheral neuropathy

**TABLE 2:** The Guillain‐Barré syndrome spectrum.

Typical GBS	Purely motor or sensory‐motor GBS, with symmetrical weakness of arms and legs and reduced/absent deep tendon reflexes
GBS variants	
Pharyngeal‐cervical‐brachial	Bilateral facial weakness ± bilateral arm paraesthesias ± weakness of the arms (no involvement of lower limbs or cranial nerves)
Paraparetic	Isolated involvement of both lower limbs
Pure sensory	Sensory symptoms and reduced/absent reflexes but no weakness
MFS spectrum (GQ1b antibody syndromes)	
MFS	Ataxia, ophthalmoplegia, areflexia – the full triad is present
	90% of patients have anti‐GQ1b antibodies
Partial MFS	Two or one out of the three clinical features of MFS
	Anti‐GQ1b antibodies found in similar percentage to MFS
GBS/MFS overlap syndrome	Features of both GBS and MFS
	Around 70% of patients have anti‐GQ1b antibodies
Bickerstaff brainstem encephalitis	Ataxia, ophthalmoplegia, hyperreflexia, altered level of consciousness
	66% of patients have anti‐GQ1b antibodies
Acute ophthalmoplegia (without ataxia)	Acute paralysis of the external (extraocular) and/or internal (intrinsic) eye muscles without ataxia. Unilateral involvement may occur [93]

Abbreviations: GBS, Guillain‐Barré syndrome; MFS, Miller Fisher syndrome.

### Ganglioside antibodies and laboratory testing

Ganglioside autoantibodies target peripheral nervous system glycosphingolipids containing sialic acid. Both the IgG and IgM isotypes are associated with GBS, whereas persistent IgM without IgG is typically found in chronic immune‐mediated neuropathies. Their presence supports a diagnosis of inflammatory (autoimmune) neuropathy, but their absence does not exclude GBS [48], does not impact on clinical management, and has no monitoring or prognostic utility. Amongst all ganglioside antibodies, anti‐GQ1b have the highest specificity (Table 3) and should be tested for in patients with suspected Miller Fisher syndrome (MFS) or GBS/MFS overlap. Patients with poor response to treatment and/or treatment‐related fluctuations should be tested for *nodal and paranodal antibodies*, targeting NF155, NF186/NF140, CNTN‐1, or Caspr‐1, which, if positive, would have diagnostic, prognostic, and therapeutic implications.

**TABLE 3:** Ganglioside antibodies in Guillain‐Barré syndrome and associated clinical utility.

Ganglioside antibody	Associated clinical findings and utility
Anti‐GM1	Elevated IgG and IgM at baseline and persistently high IgG early in the disease course correlate with poor clinical recovery [94]
	Also present in up to 80% of patients with MMN which presents with weakness (usually in the upper limbs) and conduction block on electrophysiology. This is important as MMN does not respond to plasma exchange
Anti‐GM2	May suggest CMV infection
Anti‐GD1a	Younger patients with facial weakness and prominent axonal damage [95]
	Associated with poor outcome [96]
Anti‐GT1a	Often indicate bulbar involvement in the pharyngeal‐cervical‐brachial variant of GBS [97]
Anti‐GQ1b	Highly specific for MFS, BBE, and GBS/MFS overlap syndrome

Abbreviations: BBE, Bickerstaff brainstem encephalitis; CMV, cytomegalovirus; GBS, Guillain‐Barré syndrome; Ig, immunoglobulin; MFS, Miller Fisher syndrome; MMN, multifocal motor neuropathy.

### Electrophysiology

Traditionally, slowing of conduction velocity (CV), conduction block, temporal dispersion, prolonged distal motor latencies, and delayed F waves have been associated with the acute inflammatory demyelinating polyradiculoneuropathy (AIDP) form of GBS. Conversely, patients with predominantly axonal features, namely substantially reduced compound muscle action potentials (CMAP) with relatively less pronounced conduction slowing have been classified as having the acute motor (and sensory) axonal neuropathy (AM[S]AN) forms of GBS. However, the new guidelines do not endorse any specific diagnostic criteria for these two entities. This is because the distinction between AIDP and AMAN/AMSAN does not impact on clinical management, and there is no gold standard to select among the various published criteria [2]. In general, in patients with suspected GBS, in the first week within onset of symptoms, reduced sensory nerve action potentials (SNAP) and/or CMAP would support a diagnosis of peripheral neuropathy, and the finding of absent H‐reflexes may suggest a radiculopathy. H‐reflexes are the electrophysiological correlate of reflex muscle activation after electrical stimulation of afferent sensory fibres; their absence is highly sensitive (95%–100%) [49, 50] for GBS and their presence makes a diagnosis of GBS unlikely [51, 52]. The finding of a sural‐sparing sensory pattern also facilitates early diagnosis of GBS and aids differential diagnosis with mimics [53, 54].

### Imaging

Magnetic resonance imaging (MRI) and ultrasound (USS) are not used as routine tests for the diagnosis of GBS in typical presentations but may be considered in cases where the diagnosis is uncertain. For example, MRI of the spinal cord might help distinguish a peripheral neuropathy from a myelopathy or might localise the pathology to the nerve roots. It should be noted, however, that diffuse nerve thickening on MRI is non‐specific and can indicate a wide differential of neuropathies including inflammatory, infective, infiltrative, and genetic.

## DIFFERENTIAL DIAGNOSIS OF GBS

**FIGURE 4:**
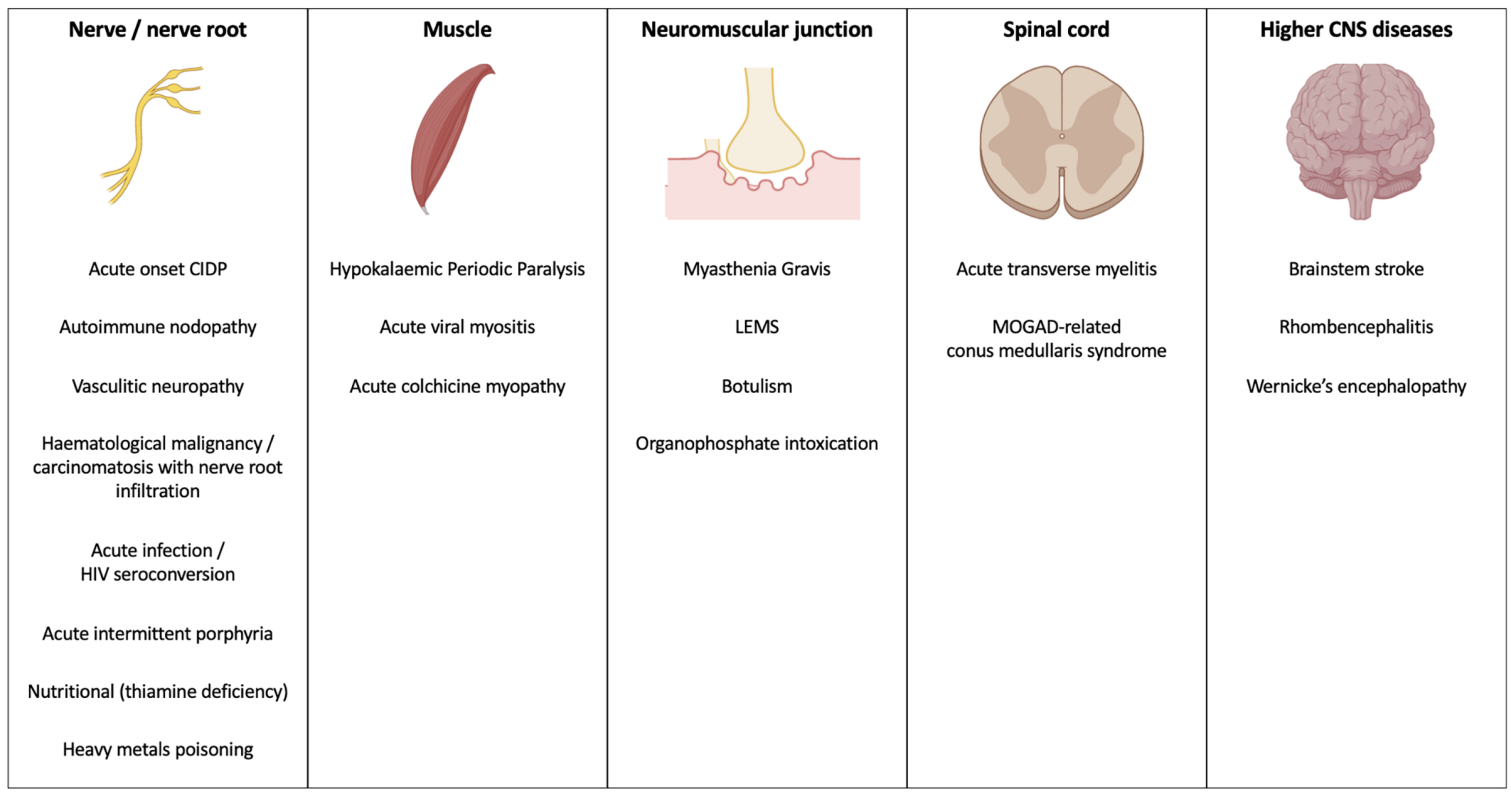
Differential diagnosis of Guillain‐Barré syndrome. CIDP, chronic inflammatory demyelinating polyradiculoneuropathy; CNS, central nervous system; LEMS, Lambert–Eaton myasthenic syndrome; MOGAD, myelin oligodendrocyte glycoprotein antibody‐associated disease.

An inflammatory neuropathy is likely if onset and progression are acute or subacute (over days or weeks), along with elevated CSF protein and improvement of symptoms upon immunosuppression or immunomodulation. GBS is most likely if the neuropathy is acute, inflammatory, and monophasic. Mimics include peripheral and central nervous system disorders, and in some cases only time will confirm or refute a diagnosis of GBS. Figure 4 summarises the differential diagnosis of GBS based on localisation.

### Peripheral nerve mimics of GBS

CIDP may have a GBS‐like acute presentation with sensory‐motor disturbance (acute CIDP or A‐CIDP). Similarly, autoimmune nodopathies (AIN) can be acute and monophasic, but often have additional features. In particular, patients with NF186 or pan‐neurofascin antibodies (against NF155, NF140, and NF186) often have a severe, GBS‐like neuropathy with cranio‐respiratory and autonomic involvement. Three or more TRFs, progression or (re)deterioration over 8 weeks from onset, or substantial sensory disturbance (including sensory ataxia) make A‐CIDP or an autoimmune nodopathy more likely. Slow disease onset (defined as >2 weeks from onset to nadir) [2] and/or significant early reduction in motor CV may also suggest an alternative diagnosis. It is important to distinguish GBS from CIDP as the latter can respond to corticosteroids, often requires long‐term or repeated treatment, and might be responsive to steroid‐sparing immunosuppression. On the contrary, autoimmune nodopathies respond poorly to conventional therapies (IVIg, PLEX, corticosteroids) and may benefit from B cell depleting immunomodulation with the monoclonal antibody rituximab if given early in the disease course [55, 56, 57].

Other peripheral nerve disorders may mimic GBS. *Vasculitic* neuropathies can be acute (although more frequently subacute, with onset over several weeks and progression over months) but are usually asymmetrical and multifocal (mononeuritis multiplex) whereas GBS, even in its variant forms (except rare monomelic cases), is symmetrical. In the early phase of disease, GBS pain is most commonly in the lower back, interscapular or radicular, and may then persist for several months, unresponsive to immunosuppression [58]. *Infective* peripheral neuropathies that can resemble GBS and present with acute flaccid paralysis include those caused by *Borrelia burgdorferi* (Lyme disease), HIV, pure paralytic forms of rabies, and diphtheric polyneuropathy. Poliovirus tends to affect the anterior horn cells of the spinal cord, and poliomyelitis‐related neuronopathy occurs in unvaccinated individuals, often after a prodromal flu‐like phase with fever and myalgia [59, 60]. Non‐polio enteroviruses, instead, typically cause diarrhoea, fever and general malaise followed, 1 or 2 weeks later, in around 20% of cases, by peripheral neuropathy [61]. HIV‐related neuropathy is usually due to seroconversion and may mimic GBS, but CSF WCC is typically high. HIV patients with low CD4 count and severe immunodepression can develop neuropathy also from opportunistic infections, and responsible pathogens include CMV, EBV, herpes simplex virus, and varicella‐zoster virus. Rarely, HIV patients develop weakness due to antiretroviral therapy or vitamin B12 deficiency [60, 62]. Poisoning with heavy metals (thallium, lead, arsenic, mercury) may cause a subacute or acute peripheral neuropathy mimicking GBS. In some cases, characteristic features such as upper limb weakness and wrist drop (lead) or pronounced sensory symptoms with ataxia (mercury) help the distinction from GBS. Similarly to GBS, peripheral neuropathy due to vitamin B1 (thiamine) deficiency may present with areflexia and progressive ascending weakness, but does not cause albuminocytological dissociation and does not have demyelinating features on nerve conduction studies. Finally, acute intermittent porphyria (AIP) can cause a GBS‐like neuropathy which usually starts with symmetrical, proximal weakness in the upper limbs, followed by involvement of the lower limbs. Less commonly, weakness begins distally with wrist or foot drop, and sensory disturbance is unusual [63]. Porphyric neuropathy can progress rapidly to tetraparesis, respiratory failure, and death if untreated, and tends to follow a prodromal phase with neuropsychiatric symptoms, abdominal pain, nausea, vomiting, and dysautonomia. Both AIP‐related neuropathy and GBS have acute onset, rapid progression, and frequent autonomic disturbance; however, porphyric neuropathy tends to be axonal on electrophysiology, lacks albuminocytological dissociation on CSF, and is unresponsive to immunotherapy.

### Neuromuscular junction disorders

*Myasthenia gravis* (MG), the most common neuromuscular junction (NMJ) disorder, causes fatigable weakness with diurnal fluctuations, preserved reflexes, and decremental response to repetitive nerve stimulation on electrophysiology. Bulbar‐onset MG and MG with positive anti‐muscle‐specific kinase (anti‐MuSK) antibodies may mimic the pharyngeal‐cervical‐brachial variant of GBS (and vice versa). *Lambert–Eaton myasthenic syndrome* (LEMS) is often paraneoplastic, causing weakness that improves with exercise, loss of reflexes, and incremental response to repetitive nerve stimulation. Although MG, LEMS, and GBS all cause muscle weakness, diurnal fluctuations and exercise‐induced effects are not typical of GBS, where reflexes are characteristically absent. *Botulism*, another NMJ disorder, presents with acute paralysis and can mimic GBS, with distinct features including descending paralysis and mydriasis. *Organophosphate intoxication* causes flaccid paralysis with a constellation of cholinergic symptoms such as diarrhoea, increased urinary frequency, lacrimation, excessive sweating and salivation, with myosis and bradycardia.

### Myelopathies

Acute spinal cord disorders can mimic GBS in the first few days following onset. Both acute transverse myelitis (inflammatory or infective) and GBS can present with acute flaccid paraparesis, sphincter disturbance with or without back pain, and reflexes can be absent in early myelopathies. Myelin oligodendrocyte glycoprotein‐associated disease (MOGAD) may present with conus medullaris syndrome mimicking GBS [64], usually with prominent sphincter disturbance at onset which is uncommon in GBS. However, a cord disorder will have a sensory level whereas GBS will usually not, and MRI (and serology where appropriate) will aid differential diagnosis.

### Myopathies

Acute muscle disorders include *hypokalaemic periodic paralysis*, some forms of *acute myositis* secondary to viral infections, and acute colchicine myopathy. Hypokalaemic periodic paralysis causes recurrent episodes of flaccid paralysis, while viral myositides, like those from influenza A, lead to rapid paralysis with myalgia, rhabdomyolysis, and fever, and may be preceded by prodromal flu‐like symptoms. Unlike GBS, hypokalaemic paralysis lacks sensory deficits, sphincter disturbance and bulbar or respiratory muscle involvement. Rhabdomyolysis and fever are common in viral myositides but rare in GBS. Acute colchicine myopathy often manifests with rapidly progressive weakness preceded by diarrhoea; however, the lack of cranial nerve involvement, raised creatine kinase, and the finding of myotonic discharges on electromyography help in differentiating it from GBS [65].

### Higher central nervous system disorders

*Brainstem stroke* may mimic GBS, especially if pontine or mesencephalic. Pontine strokes due to basilar artery occlusion (or dissection‐related subarachnoid haemorrhage or pontine compression) cause complete motor and sensory deficits affecting head, trunk, and limbs (locked‐in syndrome), and manifest with quadriplegia, anarthria, dysphagia, horizontal gaze palsy with or without preservation of vertical gaze, vertigo, and altered level of consciousness, typically with hyperacute onset. Patients with brainstem strokes have hypertonia and hyperreflexia, unusual in peripheral neuropathy. *Rhombencephalitis* can resemble GBS, but patients will have neighbourhood vertebrobasilar signs, absent in GBS. Finally, *Wernicke's encephalopathy* (WE), manifesting with the triad ataxia, eye movement abnormalities (ophthalmoplegia and/or nystagmus), and encephalopathy, can resemble MFS or BBE. However, patients with MFS do not have altered mental status or higher cortical dysfunction, and are areflexic. BBE patients may develop a similar encephalopathy but, typically, have pathologically brisk deep tendon reflexes.

## OUTCOME MEASURES IN GBS

Outcome measures in the inflammatory neuropathies are usually ordinal composite measures, and sum scores are generally used. In GBS, disease activity and clinical disability are measured using the Medical Research Council sum score (MRC‐SS), the GBS Disability Scale (GDS), formerly known as Hughes Disability Score, the Inflammatory Rasch‐built Overall Disability Scale (I‐RODS), the Inflammatory Neuropathy Cause and Treatment (INCAT) disability scale, and the Overall Neuropathy Limitation Scale (ONLS). Details of the GBS outcome measures can be found in Appendix S1.

## FLUID BIOMARKERS IN GBS

The introduction of single molecule array technology has had a profound impact on biomarker discovery, and there is mounting interest in serum and CSF biomarkers of peripheral nerve disease. Three biomarkers have been shown to be potentially useful in GBS: neurofilament light chain (NfL), peripherin, and total tau (T‐tau).

In patients with GBS, median serum levels of *NfL* on admission are significantly higher compared to age‐matched healthy controls and return to physiological levels 1 year after disease onset [66]. High serum NfL correlates with “axonal” neurophysiology and disease severity, and high baseline levels are associated with inability to run and lower I‐RODS at 1 year. NfL might be used to improve current outcome measures and prognostic models in GBS; however, it is highly non‐specific and elevated in at least 80 peripheral and central nervous system (CNS) diseases [67].

Serum levels of *peripherin*, a type III intermediate filament protein, have been shown to be significantly elevated in GBS patients with electrophysiological evidence of axonal damage [68]. Peripherin can differentiate acute nodo‐paranodal axonal injury from slowly progressive demyelination, and discriminates central versus peripheral NfL elevations by selectively rising in GBS and not in CNS disorders such as multiple sclerosis and dementia. Further work is needed to determine its contribution to clinical assessment of patients with GBS and its correlation with long‐term functional outcomes.

*T‐tau* is a microtubule‐associated protein expressed in both central and peripheral axons. Plasma but not CSF levels of T‐tau are higher in GBS compared to disease‐free and healthy controls, and plasma and CSF levels of T‐tau do not correlate [69], possibly suggesting that T‐tau found in the blood of GBS patients originates from damaged distal peripheral nerve axons. No data are currently available on the correlation of T‐tau with I‐RODS or other outcome measures, and its potential role as a diagnostic, monitoring, or prognostic biomarker remains to be determined.

## TREATMENT

Current disease‐modifying therapies in GBS include IVIg and PLEX. Corticosteroids are not recommended in GBS: intravenous methylprednisolone (IVMP) does not improve neurological outcome [70, 71] and IVMP in combination with IVIg is not beneficial [72]. Oral corticosteroids may delay recovery or cause side effects [70, 73]. Finally, a less common treatment is immunoabsorption (IA), which involves selectively removing IgG from the blood by passing separated plasma through an absorption column. Although case reports and small case series suggest that IA may be safe and effective, it is expensive and not available in all countries, and currently not recommended as first‐ or second‐line therapy for GBS [2].

### Whom and when to treat

**FIGURE 5:**
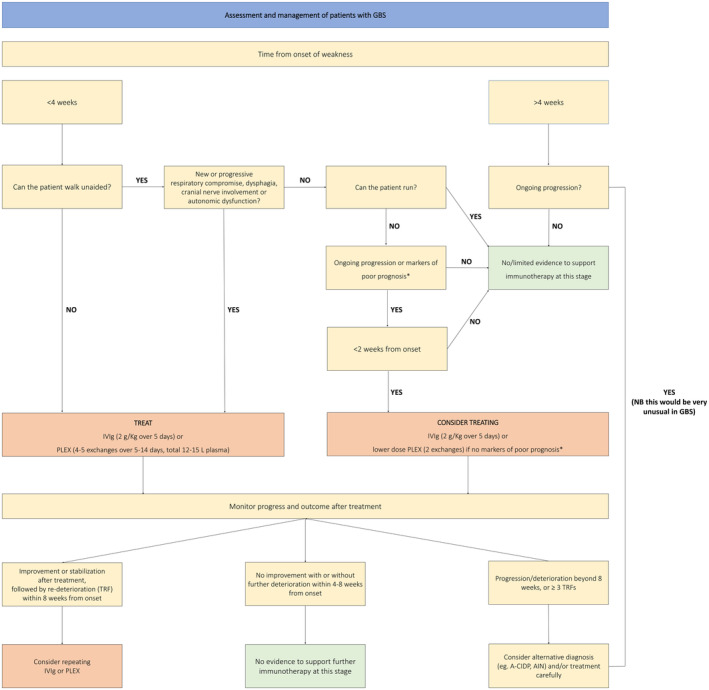
Guillain‐Barré syndrome (GBS) treatment flowchart. *Modified Erasmus GBS Respiratory Insufficiency Score (mEGRIS) ≥3/modified Erasmus GBS Outcome Score (mEGOS) ≥4. A‐CIDP, acute‐onset chronic inflammatory demyelinating polyradiculoneuropathy; AIN, autoimmune nodopathy; IVIg, intravenous immunoglobulin; PLEX, plasma exchange. [Correction added on 03 June 2024, after first online publication: Figure 5 has been corrected.]

If patients with GBS are to be treated with disease‐modifying therapy, then this should be started as early as possible within their disease course. IVIg and PLEX are thought to work by disrupting the disimmune processes driving nerve damage, preventing further nerve injury, and facilitating functional recovery. At nadir or during the recovery phase, when neuropathic processes have resolved, such treatments will no longer be of benefit. Moreover, if further nerve injury can be prevented before it occurs, and/or conduction block reversed before axonal degeneration ensues, then the extent of disability at nadir is likely to be reduced and the speed of recovery increased. Trial evidence has only shown benefit with IVIg started within 2 weeks, and PLEX started within 4 weeks, of the onset of weakness. However, it is generally felt that IVIg is also likely to be effective up to 4 weeks. Conversely, patients with mild disease (or pure MFS without GBS overlap) who are destined for a benign disease course without treatment are unlikely to benefit from such therapies even within these timeframes and may be harmed by them. Figure 5 illustrates a decision‐making algorithm for whether and when to treat based on timing and clinical features.

### IVIg versus PLEX

Head‐to‐head randomised trials directly comparing IVIg with PLEX have shown no significant difference in treatment efficacy, but IVIg is less likely to be discontinued than PLEX [74]. The choice of which therapy to offer is mostly based on pragmatic considerations and patient/clinician preference, and the latest guidelines do not support one treatment over the other [2]. Plasma exchange is not available in all centres, and IVIg might be logistically easier. IVIg is generally well tolerated but may cause thromboembolism, especially in patients with a previous thrombotic event [75]. PLEX is also well tolerated but can cause hypotension from rapid fluid shifts, fluid overload, and vasovagal episodes. Such complications may be more frequent in GBS patients who often have labile blood pressure due to autonomic nervous system involvement. Clinicians should consider suspending anti‐hypertensive therapy on treatment days. Allergic or anaphylactic reactions are rare and, when occurring, usually result from the infusion of plasma or human albumin solution. A further group of complications pertains to the vascular access, including line infection, sepsis, haematomas, venous thrombosis, and vascular damage [76].

### IVIg and PLEX regimens

The usual IVIg regimen for GBS is 2 g/kg given over 5 days. This is associated with a lower relapse (or treatment‐related fluctuation) risk compared to 2 g/kg over 2 days only [2]. Patients with severe GBS and poor prognosis should receive one course of IVIg and do *not* benefit from a second immunoglobulin dose, which increases the risk of side effects (thromboembolic and vascular) without improving neurological outcome [77, 78]. In GBS patients who can still walk unaided but are unable to run, two plasma exchanges within the first 2 weeks of disease onset are recommended. Four to five plasma exchanges over 1 or 2 weeks, with a total exchanged volume of 12–15 L, are recommended in patients who are still ambulatory but have evidence of rapid deterioration (dyspnoea, bulbar, or autonomic dysfunction). There is currently no evidence for any form of combination therapy, such as PLEX after IVIg or vice versa.

## PROGNOSTIC CONSIDERATIONS

GBS is a treatable disease and most patients ultimately recover. About 80% of patients will walk independently and over 50% return to their previous baseline within a year [79]. However, up to 10% of patients require several months of mechanical ventilation, and more than 10% are left with severe disability. Approximately 20% of patients who become ventilator‐dependent and 3–7% of all GBS patients die [79, 80, 81], usually due to sepsis, acute respiratory distress syndrome, pulmonary embolism, or cardiac arrest [82]. Negative prognostic factors include age >60 years, rapid onset of weakness (less than 7 days from symptom onset to hospital admission), severe weakness on admission, the need for mechanical ventilation, preceding diarrhoea, and features of severe neuropathy on electrophysiology.

It should be noted that death and disability rates in GBS vary significantly between developed and developing countries, largely because treatments like IVIg and PLEX are often unavailable. Additionally, inadequate rehabilitation services further contribute to disparities in functional outcomes. Small volume plasma exchange (SVPE), the repeated removal of small volumes of plasma over several days via sedimentation of patient whole blood, has been evaluated as a potential low‐cost alternative treatment [83]. Although SVPE appears feasible and safe, randomised control trials are needed to determine its clinical efficacy.

### Prediction of respiratory insufficiency

Early recognition of severe GBS is pivotal to enable prompt intervention. The Erasmus GBS Respiratory Insufficiency Score (EGRIS) is a clinical model used to predict the probability of developing respiratory failure within the first week of admission in patients with GBS. The estimated risk is based on the presence or absence of specific clinical features upon hospital admission [84]. The modified EGRIS (mEGRIS), the latest version of the model, can be used to predict respiratory failure at any point during the first 2 months from disease onset, not just in the first week. On a practical level, such risk is greater in patients with rapid disease progression in the first 4 weeks, bulbar palsy, and weakness of neck flexion and hip flexion. These patients should be monitored closely, with a low threshold for admission to the intensive care unit (ICU) if required [85, 86].

### Prediction of functional outcome

The modified Erasmus GBS Outcome Score (mEGOS) predicts the probability of being unable to walk independently at 6 months [87]. Three variables are included: age at onset, preceding diarrhoea (which may indicate *C. jejuni* infection, severe axonal damage, and poor outcome) and severity of muscle weakness (on admission and at day 7) measured using the MRC‐SS. The International GBS Outcome Study (IGOS) provides online tools based on mEGRIS and mEGOS, to estimate respiratory and motor prognosis, respectively (https://gbstools.erasmusmc.nl).

## POTENTIAL THERAPIES AND FUTURE DIRECTIONS

### Complement inhibition

New potential therapies for GBS are currently under evaluation but none are yet routinely used in clinical practice. Clinical trials of eculizumab, a humanised monoclonal antibody against the complement protein C5, have so far not provided evidence of efficacy in patients with severe GBS, either due to failure to meet the primary endpoint [88] or premature interruption [89]. At present, given lack of definitively proven efficacy, together with high costs and potential side effects, eculizumab is not recommended for the treatment of GBS, and large, randomised controlled trials are needed to establish its potential clinical utility. Inhibition of C1q factor with the monoclonal antibody ANX005 in GBS has shown preliminary evidence of efficacy (NfL reduction and improvement of GBS disability score in the first weeks after treatment) [90] and remains under evaluation.

### IgG degradation

Imlifidase is an IgG‐degrading enzyme derived from *Streptococcus pyogenes* which can cleave and inactivate all four subclasses of human IgG. IgG metabolising enzymes are able to block complement activation in vitro [91] and facilitate recovery in rabbit models of GBS with axonal injury, where they also reduce axonal degeneration [92]. In light of these findings, imlifidase is currently being evaluated in an open‐label, multicentre, phase II study as potential treatment for GBS.

## CONCLUSIONS

Guillain‐Barré syndrome is a postinfectious, immune‐mediated peripheral neuropathy. As our knowledge continues to expand, its pathophysiological mechanisms remain partially understood and many questions are yet to be answered. The focus of care should remain early diagnosis and treatment, to prevent severe axonal damage and minimise long‐term disability. Meanwhile, novel therapies and neuropathy fluid biomarkers are under ongoing evaluation and may improve clinical management in the foreseeable future.

## AUTHOR CONTRIBUTIONS

**Roberto Bellanti:** Conceptualization; writing – original draft; writing – review and editing. **Simon Rinaldi:** Supervision; conceptualization; writing – review and editing.

## CONFLICT OF INTEREST STATEMENT

On behalf of all the authors, the corresponding author states that there are no conflicts of interest.

## Supporting information


Appendix S1.

## Data Availability

Data sharing is not applicable to this article as no new data were created or analyzed in this study.
