# Supplementary material for: Guillain‐Barré syndrome: a comprehensive review
Source: Eur J Neurol. 2024 May 30;31(8):e16365. doi: 10.1111/ene.16365 (PMC11235944; doi:10.1111/ene.16365)
Supplement: Supplementary file 1 — Appendix S1. [file ENE-31-e16365-s001.doc]

**SUPPLEMENTARY MATERIAL**

**MRC Sum Score**

The MRC Sum Score (MRC-SS) was first developed to assess bedridden or ventilated patients with GBS and has then subsequently been used in a wide range of neurological diseases of the peripheral and central nervous system. It measures global muscle strength by assessing six muscle groups on both sides, with a scoring that ranges from 0 to 60. MRC-SS has served as an outcome measure in several clinical trials in patients with inflammatory neuropathies, and its interobserver agreement is 89%.^1,2^

| **Muscle** | | **MRC score** | **Sum score** |
| --- | --- | --- | --- |
| Shoulder abductors | Left |  |  |
|  | Right |  |  |
| Elbow flexors | Left |  |  |
|  | Right |  |  |
| Wrist extensors | Left |  |  |
|  | Right |  |  |
| Hip flexors | Left |  |  |
|  | Right |  |  |
| Knee extensors | Left |  |  |
|  | Right |  |  |
| Ankle dorsiflexors | Left |  |  |
|  | Right |  |  |
| **Total** | | | **__ /60** |

**I-RODS**

The Inflammatory Rasch-built Overall Disability Scale (I-RODS) is a linearly weighted scale that focuses on day-to-day life functional limitations in patients with GBS and other inflammatory neuropathies (CIDP, multifocal motor neuropathy and paraproteinemic neuropathies).^3^ Based on the Rasch model, which enables the conversion of ordinal data into an interval metric, I-RODS includes a wide range of item difficulties, from simple tasks such as “reading a newspaper” to “standing for hours” and “running”.

| **Item** | **Not possible**  **to perform**  **[0]** | **Possible, but with some difficulty**  **[1]** | **Possible,**  **with no difficulty**  **[2]** |
| --- | --- | --- | --- |
| Read a newspaper or book |  |  |  |
| Eat |  |  |  |
| Brush your teeth |  |  |  |
| Wash upper body |  |  |  |
| Wash lower body |  |  |  |
| Sit on / go to the toilet |  |  |  |
| Make a sandwich |  |  |  |
| Dress upper body |  |  |  |
| Move a chair |  |  |  |
| Turn a key in a lock |  |  |  |
| Go to general practitioner |  |  |  |
| Take a shower |  |  |  |
| Do the dishes |  |  |  |
| Do the shopping |  |  |  |
| Catch an object (e.g. ball) |  |  |  |
| Bend forward and pick up an object |  |  |  |
| Walk up a flight of stairs |  |  |  |
| Travel by public transport |  |  |  |
| Walk and avoid obstacles |  |  |  |
| Walk outdoors < 1 Km |  |  |  |
| Carry and put down a heavy object |  |  |  |
| Dance |  |  |  |
| Stand for hours |  |  |  |
| Run |  |  |  |

**GBS Disability Scale**

The Guillain-Barré syndrome Disability Scale (GDS) was first introduced in a multicentre, randomised trial of prednisolone in GBS, and has been the most widely used outcome measure in the majority of GBS trials to date. Patient disability is graded on a seven-point scale ranging from 0 (no symptoms) to 6 (dead).^4^

| **Description** | **Score** |
| --- | --- |
| Normal | 0 |
| Minor symptoms, able to run | 1 |
| Able to walk ≥10 m without assistance, unable to run | 2 |
| Able to walk 10 m with assistance | 3 |
| Bedridden or chairbound | 4 |
| Requiring assisted ventilation | 5 |
| Dead | 6 |

**INCAT**

The INCAT disability score evaluates upper and lower limb functional impairment based on the ability to perform daily life tasks.^5–7^ Each category is scored from 0 to 5 and the total INCAT score ranges between 0 and 10, with higher scores indicating more severe impairment.

**Arm Disability**

1. No upper limb problems
2. Symptoms, in one or both arms, not affecting the ability to perform the following functions: doing all zips and buttons, washing or brushing hair, using a knife and fork together, handling small coins
3. Symptoms, in one arm or both arms, affecting but not preventing any of the previously mentioned functions
4. Symptoms, in one arm or both arms, preventing one or two of the previously mentioned functions
5. Symptoms, in one arm or both arms, preventing all the functions listed, but some purposeful movements still possible
6. Inability to use wither arm for any purposeful movement

**Leg Disability**

1. Walking not affected
2. Walking affected, but walks independently outdoors
3. Usually uses unilateral support (stick, single crutch, one arm) to walk outdoors
4. Usually uses bilateral support (sticks, crutches, frame, two arms) to walk outdoors
5. Usually uses wheelchair to travel outdoors, but able to stand and walk a few steps with help
6. Restricted to wheelchair, unable to stand and walk a few steps with help

**ONLS**

The Overall Neuropathy Limitation Scale (ONLS) is often used to assess patients with GBS and was derived from the Overall Disability Sum Score (ODSS), which was the first scale designed to evaluate arm and leg functional limitations in patients with immune-mediated peripheral neuropathies, including GBS, CIDP and paraprotein-associated neuropathies.^8^ Although the Arm Scale is identical in ODSS and ONLS, the latter was modified to include climbing stairs and running. The Arm Scale score ranges from 0 to 5, the Leg Scale from 0 to 7, and the total ONLS is 0-12.

| **Arm Scale** | | | | |
| --- | --- | --- | --- | --- |
| Does the patient have any symptoms in their hands or arms, e.g. tingling, numbness or weakness? (if no, go to legs section) | Yes | | No | |
|  | **Not affected** | **Affected but not prevented** | | **Prevented** |
| Wash and brush their hair |  |  | |  |
| Turning a key in a lock |  |  | |  |
| Using knife and fork / spoon / other |  |  | |  |
| Do or undo buttons or zips |  |  | |  |
| If all above functions are prevented, can the patient make purposeful movements with their arms or hands? | Yes | No | | Not applicable |

**Arm Score**

1. Normal
2. Minor symptoms in one or both arms but not affecting any of the functions listed
3. Disability in one or both arms affecting but not preventing any of the functions listed
4. Disability in one or both arms preventing at least one but not all functions listed
5. Disability in both arms preventing all functions listed but purposeful movement still possible
6. Disability in both arms preventing all purposeful movements

| **Leg Scale** | | | |
| --- | --- | --- | --- |
|  | Yes | No | Not applicable |
| Does the patient have difficulty running or climbing stairs? |  |  |  |
| Does the patient have difficulty with walking? |  |  |  |
| Does their gait look abnormal? |  |  |  |
| How do they mobilize for about 10 meters? usually get around for about 10 meters (choosing one of the four options below)? |  |  |  |
| - Without aid |  |  |  |
| - With one stick or crutch or holding to someone’s arm |  |  |  |
| - With two sticks or crutches or one stick or crutch and holding to someone’s arm |  |  |  |
| - With a wheelchair |  |  |  |
| - If they use a wheelchair, can they stand and walk a few steps with help? |  |  |  |
| If they cannot walk, are they able to make some purposeful movements of their legs (e.g. reposition legs in bed)? |  |  |  |
| Does the patient use ankle-foot orthoses? |  |  |  |

**Leg Score**

1. Normal
2. Walking or climbing stairs or running affected, but gait does not look abnormal
3. Walks independently but gait looks abnormal
4. Requires unilateral support to walk 10 metres (stick, single crutch, one arm)
5. Requires bilateral support to walk 10 metres (sticks, crutches, crutch and arm, frame)
6. Requires wheelchair to travel 10 metres but able to stand and walk 1 metre with the help of one person
7. Restricted to wheelchair, unable to stand and walk 1 metre with the help of one person, but able to make some purposeful leg movements
8. Restricted to wheelchair or bed most of the day, unable to make any purposeful movements of the legs

**Further reading**

1. Turan Z, Topaloglu M, Ozyemisci Taskiran O. Medical Research Council-sumscore: a tool for evaluating muscle weakness in patients with post-intensive care syndrome. *Crit Care*. 2020;24(1):562. doi:10.1186/s13054-020-03282-x

2. Kleyweg RP, Van Der Meché FGA, Schmitz PIM. Interobserver agreement in the assessment of muscle strength and functional abilities in Guillain-Barré syndrome. *Muscle Nerve*. 1991;14(11):1103-1109. doi:10.1002/mus.880141111

3. van Nes SI, Vanhoutte EK, van Doorn PA, et al. Rasch-built Overall Disability Scale (R-ODS) for immune-mediated peripheral neuropathies. *Neurology*. 2011;76(4):337-345. doi:10.1212/WNL.0b013e318208824b

4. Hughes RA, Newsom-Davis JM, Perkin GD, Pierce JM. Controlled trial prednisolone in acute polyneuropathy. *Lancet Lond Engl*. 1978;2(8093):750-753. doi:10.1016/s0140-6736(78)92644-2

5. Randomized controlled trial of intravenous immunoglobulin versus oral prednisolone in chronic inflammatory demyelinating polyradiculoneuropathy - PubMed. Accessed March 22, 2024. https://pubmed.ncbi.nlm.nih.gov/11506402/

6. Vanhoutte EK, Faber CG, Merkies ISJ, PeriNomS study group. 196th ENMC international workshop: Outcome measures in inflammatory peripheral neuropathies 8-10 February 2013, Naarden, The Netherlands. *Neuromuscul Disord NMD*. 2013;23(11):924-933. doi:10.1016/j.nmd.2013.06.006

7. Incat disability score: A critical analysis of its measurement properties - Breiner - 2014 - Muscle & Nerve - Wiley Online Library. Accessed October 28, 2023. https://onlinelibrary.wiley.com/doi/10.1002/mus.24207

8. Graham RC, Hughes RAC. A modified peripheral neuropathy scale: the Overall Neuropathy Limitations Scale. *J Neurol Neurosurg Psychiatry*. 2006;77(8):973-976. doi:10.1136/jnnp.2005.081547
